# “I just finally wanted to belong somewhere”—Qualitative Analysis of Experiences With Posting Pictures of Self-Injury on Instagram

**DOI:** 10.3389/fpsyt.2020.00274

**Published:** 2020-04-21

**Authors:** Rebecca C. Brown, Tin Fischer, David A. Goldwich, Paul L. Plener

**Affiliations:** ^1^ Department of Child and Adolescent Psychiatry and Psychotherapy, University of Ulm, Ulm, Germany; ^2^ Independent Researcher, Berlin, Germany; ^3^ Department of Child and Adolescent Psychiatry, Medical University of Vienna, Vienna, Austria

**Keywords:** non-suicidal self-injury, self-harm, social media, qualitative study, online, motivation

## Abstract

Non-suicidal self-injury (NSSI) is a common phenomenon among adolescents, but is often not disclosed due to fear of stigmatization. Social media is frequently used to publish photos of NSSI and share experiences with NSSI. Objectives of this study were to find out more about the motivation for publishing NSSI content and to investigate the effect that sharing this content on social media has on young people. In the current study, we interviewed N=59 participants (mean age = 16.7 years [SD = 1.2 years]; 72.9% female), who had all posted NSSI content within the past month on the social media platform Instagram. Semi-structured interviews were conducted *via* the Instagram messaging app. Interviews were analyzed qualitatively, assisted by the Software Atlas.ti 7. Participants were asked about their motivation for and their experiences with posting NSSI content online. Motivations for posting pictures online were mainly social (connecting, disclosure, communicating), while self-focused reasons like documenting NSSI or recovery were also mentioned. All participants reported having received positive reactions (being offered help, connecting, receiving empathy), as well as negative comments (harassment, being misunderstood) to their own NSSI content by other Instagram users. Participants' reactions to other users' NSSI content on Instagram was often identification with the content or being triggered, but also wanting to offer help or sometimes even being deterred from NSSI. None of the participants mentioned successful referral to professional help through their online NSSI activity. One target for future interventions could therefore be social media, or other online platforms, where adolescents might be more easily reached. Mental health practitioners should be aware of their clients' online activity and encourage reflection upon positive and negative effects of viewing or sharing NSSI content online.

## Introduction

Non-suicidal self-injury, defined as the intentional damage to the surface of the body (e.g. cutting, burning, or bruising) without suicidal intent (1), is a major concern among adolescents (2). Despite high prevalence rates, affected adolescents often do not come to the attention of mental health professionals (3). Disclosure is often prevented by shame, stigma, or concern for others (4) and adolescents often prefer peer versus professional support (5). This preference might be nurtured by stigma perceived from family, friends, or even professionals (6, 7). Furthermore, school staff, who might be the first adults to notice NSSI, often report a lack of knowledge and skills concerning NSSI and may hold negative attitudes (i.e. viewing NSSI as a manipulative and attention seeking behavior) towards students presenting with NSSI (8–11).

Not only among adolescents with NSSI, but also in the general population, social media networks and online messaging services, like Instagram or WhatsApp have become highly important means for social interactions among youth (12). Given the highly sensitive nature of NSSI and the generally frequent use of online media in this age group, many adolescents and young adults with NSSI rather turn to the Internet to find information and to receive validation and social support (13–15). A recent study on Internet use among adolescents with NSSI (N=142, mean age around 14 years) found that adolescents with more recent NSSI reported higher levels of social support-seeking Internet use (and sharing NSSI content), rather than adolescents with NSSI in the past or no lifetime NSSI (16).

Sharing NSSI content on social media platforms like Instagram or YouTube has been the subject of a number of studies [for review see (15)]. Especially the possibility of disclosing a history of NSSI anonymously might be of note, given the fear of stigmatization in “real life” (13, 17). A recent study (18), analyzing all pictures with the most prominent NSSI related hashtags in Germany on Instagram in a four week period in April 2016 (N=32,182) showed a large number of pictures explicitly portraying wounds caused by NSSI (N=2,826). The number of comments (and therefore attention) a picture received was significantly associated with the severity of the wound being portrayed. The tone of comments was mainly empathetic or offering help, while only a minor percentage of comments was abusive (18). This is in line with the “double-edged sword” described by Lewis & Seko (15), stating that social media activities regarding NSSI are beneficial on the one hand (i.e. reducing social isolation, disclosure, reducing NSSI urges, and recovery encouragement), but also potentially harmful on the other hand (i.e. triggering, NSSI reinforcing, stigmatization of NSSI).

Motives for posting NSSI material online have been explored in previous studies. One study used open-ended online questions (13), whereas in another one, motives were examined through analysis of what youth posted on a popular online forum (5). To date, only one qualitative interview study with N=17 participants (19) investigated motivations for creating NSSI content online. Participants had posted NSSI content in an online community focusing on NSSI (“Self-Injury.net”). NSSI content was both textual (i.e. poetry, short stories, and essays) or visual (i.e. drawings, photographs). The study found two major motives, being self-oriented motivation (to reflect NSSI experience, to express self, to reduce self-destructive urges) and social motivation (to raise social awareness, to help others, to seek out peers).

The current study adds to this literature by way of using interviews, focusing on NSSI imagery, and considering the highly popular social network—Instagram.

Aims of the current study were (1) to qualitatively assess the motivation in young people with NSSI to share pictures of their NSSI wounds on a large social media platform (Instagram), (2) to gain more insight in the reactions adolescents with NSSI have to viewing NSSI pictures online, and (3) how those adolescents perceive comments on (their own) NSSI pictures.

## Methods

### Data Collection

Participants were identified from a larger data-set investigating the occurrence of non-suicidal self-injury (NSSI) on Instagram (18). All pictures and user accounts associated with the 16 German hashtags most commonly related to featuring pictures of NSSI wounds (i.e. #ritzen, “#cutting”) were downloaded at an hourly rate during four weeks in April 2016. For details on how those hashtags were identified see Brown et al. (18). After those four weeks of Instagram data collection, a total of N=100 randomly chosen users from this data-set (who had all posted at least one picture of NSSI on Instagram) were approached *via* Instagram messenger and asked if they were willing to participate in an interview-based study. Participants were also asked if the wounds or scars portrayed in the posted pictures were results of their own NSSI. If they agreed, participants were included in the study. Interviews were conducted on Instagram messenger using chats, which allowed participants to stay anonymous. The interviews were semi-structured and consisted of 33 questions about the participants' experiences with NSSI and suicidality on Instagram. Additionally, socio-demographic variables (i.e. gender, age) were assessed. In the current study, the questions “What was the reason you first posted pictures online?”, “What is your general intention of posting pictures on Instagram?”, “What reactions did you get regarding the pictures you posted?”, “Do you get the same amount of attention for all pictures you post, or are the pictures portraying NSSI any different?”, and “How do you feel when viewing NSSI pictures posted by other Instagram users?”.

### Ethics

Procedures contributing to this work comply with the ethical standards of the relevant national and institutional committees on human experimentation and with the Helsinki Declaration of 1975, as revised in 2008. All procedures involving human subjects were approved by the IRB of the Ulm University. Written informed consent *via* Instagram messenger was obtained from all subjects. Participants were informed about the purpose and risks of the study and about the use of their data for anonymous scientific publication *via* Instagram messenger. They agreed to those terms in written form *via* the messenger. Participants also consented to the publication of their indirectly identifiable data. All participants had to indicate to be over the age of 16. In case of acute suicidality, they were provided with emergency help advice (nation-wide telephone numbers) and were offered to talk to a trained child and adolescent psychotherapist (first author of this manuscript) on the phone or *via* Instagram messenger. None of the participants made use of this option.

### Participants

Of the N=100 users on Instagram who were initially approached, N=64 agreed to participate in a qualitative interview regarding their experiences with NSSI on Instagram, of which N=59 completed the interview. Data of these 59 participants (of which n=9 did not want to answer questions on socio-demographic variables, but completed the interview) are presented in the present paper. Participants were on average 16.7 years old (SD=1.2 years) and 72.9% were female. Most participants were students (67.8%), 11.9% were trainees, and 5.1% were unemployed. All adolescents had engaged in NSSI in the past month and had posted pictures of their NSSI wounds on Instagram.

### Qualitative Data Analyses

The software ATLAS.ti 7 was used to aid with the coding process. Qualitative analyses were conducted according to Mayring (20). In a first step, responses to the questions in the interviews were paraphrased (i.e. *Part. 12: “Hm, a few days before [first posting a picture] I had had a relapse (I had been clean for a year) and, so I searched Instagram, explicitly for it [NSSI content], and then I thought … maybe it'll help, just coming out with it. I mean, in my normal life I have to hide it, so why not post it here anonymously and talk about it”—Paraphrase: “Initial reason for posting NSSI pictures: Thinks anonymous self-disclose might be helpful, keeps NSSI secret in real life.”* In a second step, an abstraction level was defined and paraphrased content was further condensed according to this level (Condensed paraphrase: “initial reason for posting NSSI pictures: anonymous self-disclosure”). In a third step, paraphrases with the same meaning were condensed and only paraphrases with central significance for the material were kept (i.e. categories “initial reason for posting NSSI pictures: anonymous self-disclosure” and “initial reason for posting NSSI pictures: self-disclosure” were combined). In a fourth step, paraphrases with similar content were further condensed and, if needed, rephrased (i.e. categories “social connection,” “being part of a community,” “raising awareness” were combined into “social reasons for posting NSSI pictures”). Two independent raters were thoroughly trained. First, both raters rated one interview script under close supervision. Afterwards, both raters independently rated a sample of three scripts under continuous supervision. After an interrater-reliability of kappa=.81 was achieved, they continued to rate the remaining manuscripts. Interrater-reliability was kappa=.86 for all interviews (before disagreements on ratings were resolved). Whenever there was a disagreement between two ratings, an agreement was found between both raters, supervised by one of the authors of the paper [RB]. Consensus on disagreements was established in regular meetings throughout the coding process.

## Results

Results are organized by categories of replies to the interview questions. All frequencies are presented in **Table 1**.

**Table 1:** Frequencies of categories of replies to interview questions.

Category	Sub-category	N (%)
Reasons for posting NSSI pictures		
Social reasons	Social connection - Initial (first) posting - General posting	
		34 (57.6%) 23 (39.0%)
	Self-disclosure - Initial (first) posting - General posting	
		9 (15.3%) 13 (22.0%)
	Raising awareness - Initial (first) posting - General posting	
		6 (10.2%) 3 (5.1%)
	Helping others - Initial (first) posting - General posting	
		0 5 (8.5%)
	Imitation - Initial (first) posting - General posting	
		4 (6.8%) 0
Self-orientated reasons	Documentation of NSSI - Initial (first) posting - General posting	
		9 (15.3%) 7 (11.9%)
Reaction to NSSI pictures posted by others	Differentiated reaction to NSSI pictures by severity of depicted injury	
	Identification	21 (35.6%)
	Trigger	18 (30.5%)
	Feeling need to help others	15 (25.4%)
	Feeling indifferent	10 (16.9%)
	Motivation to end own NSSI	7 (11.9%)
Others' reaction to own NSSI pictures		
Positive reactions	Offering help	29 (39.2%)
	Expressing Empathy	16 (27.1%)
	Compliments	5 (8.5%)
Negative comments	Being harassed	24 (40.7%)
Amount of reaction to NSSI pictures	More attention than other pictures	32 (54.2%)
	Less attention than other pictures	1 (1.7%)
	No difference between pictures	15 (25.4%)
	Don't know	11 (18.6%)

N, number of participants; %, percent; NSSI, non-suicidal self-injury. As this was a qualitative study, multiple themes emerged for some participants towards one question.

### Social Reasons for Posting NSSI Pictures

Most of the participants (N = 36, 61%) stated social reasons for initially posting their first picture online, or for continuing to post pictures online (N = 31, 52.6%).

#### Reason for Posting NSSI Pictures: Social Connection

Reasons of social connection, i.e. belonging to a group were mentioned frequently. Many participants expressed that they felt understood by other Instagram users much more than by family members or friends in real life.

Part. 54: “I just finally wanted to belong somewhere”

#### Reason for Posting NSSI Pictures: Self-Disclosure

Other participants mentioned that they mainly posted pictures for reasons of self-disclosure. Several participants mentioned the fact that they felt safe talking about their NSSI in the anonymity of the Internet.

Part. 12: “Hm, a few days before [first posting a picture] I had had a relapse (I had been clean for a year) and, so I searched Instagram, explicitly for it [NSSI content], and then I thought … maybe it'll help, just coming out with it. I mean, in my normal life I have to hide it, so why not post it here anonymously and talk about it”

Part. 13: “(…) I just wanted ANYONE to realize how badly off I was, how urgently I needed help, because no one in my real life noticed.”

#### Reason for Posting NSSI Pictures: Raising Awareness

Another social reason was to raise awareness of reasons for self-injury and use NSSI for social signaling.

Part. 32: “…Because I wanted to show my environment that not everything is looking bright.”

Part: 1: “With my pictures and my thoughts I want to open other peoples' eyes. I want to show them that there is no use to engage in self-injury”

#### Reason for Posting NSSI Pictures: Helping Others

Helping others with NSSI was another major social reason. Many participants mentioned that they offered help to other Instagram users and talked to them privately using the Instagram messenger. The main reason (48% of participants mentioned this) for offering help was because it felt good to be of help. Another 37.8% mentioned to be offering help for purely altruistic reasons.

Part. 59: “I don't know. I post it [NSSI picture], others contact me and we talk … I mean not about myself but in my comments I mention that if others feel the same way they can contact me and then I always try to help them”

#### Reason for Posting NSSI Pictures: Imitation

The third major theme that emerged for initially starting to post pictures of NSSI was imitation (N=4, 6.8%). Participants mentioned that they had been following other NSSI-pages and had, after some time, decided to start posting their own pictures. Overall, 24 participants (40.7%) stated to have been following NSSI-pages for several months before starting to post pictures themselves. However, only four of them mentioned this to be the main reason to have started to post pictures themselves.

Part. 50: “I had been following pages which uploaded such pictures for a while and then thought about it and then also started posting pictures”

### Self-Orientated Reasons for Posting NSSI Pictures (Documentation of NSSI)

Apart from social reasons, some participants started posting pictures for documentation (N=9, 15.3%). Some stated to use it as a diary without any further given intent, whereas some participants clearly stated to try and document their recovery.

Part. 56: “I wanted to document the self-injury somewhere, to get an overview on how it develops over time.”

Part. 21: “I'm not seeking for attention, it is more like a diary, so I can see if I'm making progress, how long I've managed without it. If people are interested, they can follow me, if they are bothered by it, they should leave.”

### Reaction to NSSI Pictures Posted by Others

Interestingly, all participants only talked about pictures of cuts. Other methods of NSSI were not mentioned.

#### Differentiated Reaction to NSSI Pictures by Severity of Depicted Injury

Some participants differentiated their own reaction to NSSI pictures by the severity of the wound depicted. Generally, superficial cuts did not evoke major emotions, most participants identified with medium cuts, while very deep cuts usually led to being repulsed or gaining motivation to end their own self-injury.

Part. 47: “1…very superficial cuts, like scratches … I'm thinking “how cute is this, that's how I started off”, etc….2. my category, where the cuts are gaping … I compare myself with those and if they are a little bit worse than mine, then I find them also pretty (I generally think my scars are pretty)….3. Extreme cuts, where you can see the flesh and I mean those where the arm is really deformed, I often/mainly find those repulsing, but there are phases where I find them beautiful.”

Part. 14: “It varies. But if there are pictures of very deep wounds it repulses me sometimes and keeps me from cutting this deep. Sometimes these pictures are “hardcore”. But other pictures trigger me a lot, so I want to self-injure again and pictures of very superficial wounds don't trigger much emotion. In those cases I am rather “proud” of my wounds if they are deeper than those of others.”

#### Reaction to NSSI Pictures: Identification

The most common reaction to other users' NSSI pictures was to identify with them (N=21, 35.6%) and compare one's own wounds with those pictures.

Part. 20: “I find myself in most pictures”

#### Reaction to NSSI Pictures: Trigger

Around a third of all participants reported to be triggered by online NSSI content (N=18, 30.5%).

Part. 52: “Videos have made me cut myself even though I was feeling good at the time”

#### Reaction to NSSI Pictures: Feeling the Need to Help Others

Another reaction to NSSI pictures online was feeling the need to help others (N=15, 25.4%).

Part. 15: “…most of the time I understand why they did it and am trying to help them”

#### Reaction to NSSI Pictures: Feeling Indifferent

On the contrary, some participants mentioned to feel indifferent when looking at NSSI pictures (N=10, 16.9%).

Part. 11: “[I feel] nothing, I just look at them and keep searching”

#### Reaction to NSSI Pictures: Motivation to End NSSI

Another group of participants also mentioned to gain motivation to end their self-injury by looking at NSSI pictures online (N=7, 11.9%). However, all participants mentioned that this was a rather momentary effect and had not helped them ending NSSI completely.

Part. 2: “But through those pictures you see what it [NSSI] has done to people and it keeps you from cutting sometimes”

### Others' Reaction to Own NSSI Pictures

#### Other's Reaction: Both Negative and Positive

All participants mentioned to have received positive as well as negative reactions to their pictures. Most participants seemed to rate positive comments as quite valuable and felt a connection with other Instagram users. They seemed to be able to ignore negative comments or to just take them into account. However, N = 23 participants mentioned to feel sad or angry after having read negative comments.

Part. 1: “I got a lot of feedback. Some said I should kill myself and cut myself to death, that I was only doing it for attention. Others said that they were there for me if ever I needed someone to talk.”

Part. 37: “It varies. Some wear you down, e.g. “how sick can someone be?”. Or that I should die. But some people also commented, e.g. “you can get out of it”. Or “we believe in you”.

#### Positive Reactions: Offering Help

The main positive reaction mentioned was being offered help (N=29, 39.2%). However, of those participants who had been offered help, only 15.2% thought those conversations had actually been helpful. Most participants said that talking to others with similar problems had felt good, but did not change anything in the long term. Reasons were that they realized they had problems that couldn't be solved *via* Instagram messenger, that no actual help was offered, or that the other person was not really interested in helping in the end. Many participants also mentioned that they stopped the conversations themselves, as they felt they were leading nowhere. No participant mentioned to have been offered information on, or recommendation for professional help.

Part. 47: “a lot of them say that they are there for you. A lot of them offer you to talk to them”

#### Positive Reactions: Empathy

The second positive reaction were empathetic comments (N=16, 27.1%).

Part. 13: “…Empathy and comments by people who were in the same situation”

#### Positive Reactions: Compliments

Receiving compliments for their pictures or their injuries was also mentioned by some participants (N=5, 8.5%).

Part. 9: “some thought it was cool”

Part. 53: “concerning the pictures that only showed scars people told me how beautiful my scars were”.

#### Negative Reactions: Being Harassed

Negative reactions were being harassed, being told to commit suicide or only seeking attention (N=24, 40.7%).

Part. 50: “90% of the comments were negative and abusing me and wearing me down, although I was already feeling down”.

### Amount of Reaction Received for NSSI Pictures as Compared to Other Pictures

Most participants (N=32, 54.2%) stated to have received more attention for pictures explicitly showing NSSI wounds than for other pictures. N=15 participants (25.4%) did not see a difference in attention and one participant (1.7%) thought to have received less attention for NSSI pictures than for other pictures. The rest of the participants was not sure.

## Discussion

This is the first study to have interviewed adolescents with NSSI on their motivation for and their experiences with posting NSSI content on Instagram by using a semi-structured chat based interview.

Regarding the motivation for posting pictures online, results of this study validate those from a smaller sample of participants posting NSSI content in a self-injury online community (19). Two main themes emerged, being social and self-oriented motivation. However, in the current study, social reasons were mentioned much more often than self-oriented reasons.

Many participants mentioned to post NSSI pictures because they wanted to be part of a group or a community, to “belong.” In Maslow's hierarchy of needs, “belongingness” plays a central role (21). Furthermore, one central developmental task during adolescence is to form interpersonal relationships with peers. On the other hand, the role of peer- and family related loneliness in association with NSSI has been described in several studies (22, 23). Furthermore, social exclusion and bullying have shown to be risk-factors for NSSI (24, 25), with emerging neurobiological evidence of adolescents with NSSI to be more sensitive to social exclusion than their peers (26). The need to belong might therefore be one key-factor for adolescents posting NSSI content online. It is therefore not surprising that many of the adolescents interviewed in the current study mentioned the positive sense of community between self-injurers on Instagram. This is also in line with recent reviews of the literature [e.g., (5, 15)] on online communication about NSSI. These motives reported in prior work exploring different social platforms and types of online-activity therefore also have relevance for posting pictures on Instagram, a large and contemporary social media platform.

Interestingly, the benefit of being part of this community and receiving positive and empathetic comments seemed to outweigh the negative comments and harassment participants reported quite frequently. Surprisingly, when systematically analyzing comments that were posted under NSSI content on Instagram (in the same sample from which the participants in the current in-depth study were drawn from), we found that only a very minor amount of comments (6.5%) were actually abusive (18). Over-reporting of negative comments in the current study might be an indication of those comments having a very high emotional impact on affected adolescents and might pose a risk-factor for worsening of mood, increased feelings of rejection and therefore possibly increasing or persisting NSSI. On another note, in a recent experimental study, participants who were exposed to hopeful comments under NSSI content on YouTube showed a significant increase in their attitudes towards recovery, while negative comments did not show a negative effect (27). As adolescents keep posting pictures on social media despite the risk of being verbally harassed, the effect of positive feedback and social support (with possible positive effects on recovery) should be further explored.

In line with the need of being part of a community, the theme of wanting to help others emerged frequently. Participants mentioned not only to be posting pictures in order to help others (i.e. to discourage them from engaging in NSSI or to get their attention to then offer help), but also to feel the urge to help peers when seeing their NSSI content. Being offered help was also one of the most frequently positive effects of posting pictures online mentioned in this study. This is in line with research showing that adolescents with recent NSSI show higher levels of support-seeking internet use than adolescents with less recent or no NSSI (16) and adolescents preferring help offered by peers over family members or health care professionals (28). Also, many adolescents in the current study mentioned self-disclosure in an anonymous space to be one reason for posting NSSI pictures. The anonymity of the Internet is possibly a prominent factor, helping to overcome stigma or feelings of shame, which prevents adolescents from seeking help and disclosure in the “real world” (6, 7, 28). However, participants also mentioned that help offered by peers on Instagram was rarely helpful and did not lead to a reduction of their NSSI. One way to counter-act this problem might be the implementation of Online-therapies that guarantee anonymity. In recent years, several approaches using the Internet to facilitate interventions for NSSI have been evaluated. For example, one online intervention for NSSI is currently being evaluated in a large multi-center randomized controlled trial (RCT) (29). However, many adolescents might be reluctant to talk to mental health professionals in general, face-to-face or online. Therefore, another approach could be the use of mobile applications (apps). Franklin et al. (30) reported effectiveness for the “Therapeutic Evaluative Conditioning App” in three RCTs. Furthermore, Hooley et al. (31) have described the potential of different online writing assignments in a recent RCT.

Another interesting point that emerged from the interviews was that participants stated on the one hand to be posting pictures in order to deter peers from NSSI or to simply keeping a “diary.” On the other hand, many participants reported to feel triggered when viewing NSSI content online. This is of special note, as although it has often been put forward that NSSI material on social media can be triggering [e.g., (32)], so far there is little quantitative evidence to support this notion. Healthcare professionals should therefore discuss and reflect upon the online behavior with their clients, in order to make them aware of the possibly triggering effects on others and themselves. Guidelines for healthcare professionals on this topic have been previously published (33, 34).

The possibility of social reinforcement regarding NSSI behavior by receiving attention for NSSI pictures has been mentioned previously (15, 18, 35). The current study adds evidence of this possible risk, by participants stating to be aware that their pictures portraying NSSI received more attention than their other pictures. As a major concern, we found in a previous study (18) that pictures showing more serious wounds also generated more attention, with the possible risk of adolescents posting pictures of more serious wounds in order to get more attention. Another concern arising from the answers of participants in the current study is the one of social contagion. Many participants mentioned to have been following pages with NSSI content first, before starting to post their own NSSI pictures. As a first step to prevent such possible social reinforcing, as well as potential contagious, and triggering effects, Instagram has recently taken action and has banned NSSI related hashtags from their platform. While this is a step towards preventing social contagion, banning content from a social media network can be discussed controversially. First merely blocking hashtags is not a solution (i.e. if #self-injury is blocked, new hashtags such as #self-injuryyy can be created (36). Therefore, content has to be manually checked by commercial content moderators, which poses a tremendous workload. More importantly, if one social media platform is not accessible, content can just as easily be published on other social media platforms or homepages. Furthermore, the current study and other previous studies have shown that having an online community to exchange about NSSI and related problems can be helpful to some individuals. Taking this opportunity away without offering a (better) alternative, is not the most viable solution.

Despite being able to recruit a relatively large sample size for a qualitative study, results might still be selective, due to the nature of the study. Although participants were chosen and contacted randomly, there may still have been a self-selection bias. Furthermore, results of this study cannot be replicated, as Instagram has since changed its policy on NSSI content.

Taken together, this study provides further evidence for the motivation and experiences of posting NSSI content online. The role of social belonging and reinforcement, as well as the search for help online in adolescents with NSSI was clearly shown by results of the current study. Furthermore, risks of triggering content and social contagion were mentioned by participants. Future research on how to use those factors to help adolescents with NSSI, i.e. by implementing online therapies, mobile applications, or using the commenting function on social media to instill attitudes of hope for recovery and professional help seeking are warranted.

## Data Availability Statement

The datasets for this article are not publicly available because they consist of qualitative interviews that are not completely anonymous due to the nature of some answers given and connections to the (online) identity of participants might be possible. Requests to access the datasets should be directed to RB, rebecca.brown@uniklinik-ulm.de.

## Ethics Statement

The studies involving human participants were reviewed and approved by University of Ulm. Written informed consent *via* Instagram messenger was obtained from all subjects. Participants were informed about the purpose and risks of the study and about the use of their data for anonymous scientific publication *via* Instagram messenger. They agreed to those terms in written form *via* the messenger. Participants also consented to the publication of their indirectly identifiable data.

## Author Contributions

RB and PP take responsibility for the integrity of the work as a whole, from inception to published article. PP, TF, and RB designed the study; TF, DG, and RB acquired the data. RB analyzed and interpreted the data. RB and PP drafted the manuscript and all authors revised it critically for important intellectual content. All authors approved the final version to be published.

## Funding

This work was supported by a grant from the Volkswagen Foundation.

## Conflict of Interest

PP has received research funding from Lundbeck and Servier. 

The remaining authors declare that the research was conducted in the absence of any commercial or financial relationships that could be construed as a potential conflict of interest.
